# Quality of Life and Psychological Burden of Parents of Children, Adolescents, and Young Adults with Type 1 Diabetes: A Cross-Sectional Study during the Lockdown Period of COVID-19

**DOI:** 10.3390/nursrep12030055

**Published:** 2022-08-06

**Authors:** Nikolaos Rikos, Andreas Mpalaskas, Maria Fragiadaki, Chara Frantzeskaki, Anna Kassotaki, Manolis Linardakis

**Affiliations:** 1 School of Health Science, Department of Nursing, Hellenic Mediterranean University, Heraklion 71410, Greece; 2 Department of Social Medicine, Faculty of Medicine, University of Crete, Heraklion 70013, Greece

**Keywords:** perceived quality of life, psychological burden, type 1 diabetes, parents, children

## Abstract

The current study aimed to investigate how parents of children, adolescents, and young adults with DM1 perceived quality of life and psychological burden during the lockdown period of COVID-19. A cross-sectional study was carried out on 110 parents in Greece in spring 2021. Perceived quality of life was measured using the Parent Diabetes Distress Scale, and psychological burden was measured using the Spielberger State/Trait Anxiety Inventory, and both were assessed with correlational analysis. Overall, 79.1% of the parents were females ,while the mean age of all was 44.4 years (±5.8). PDDS was found to be moderate (mean 2.42 ± 0.76): 63.6% of respondents had moderate/high distress. The highest mean score was for Teen Management Distress and the lowest for Healthcare Team (3.02 vs. 1.49, *p* < 0.001). STAI was found to be moderate to high, with a higher mean score for state versus trait anxiety (49.8 vs. 48.0, *p* = 0.006). Increased distress or poorer parents’ quality of life was related with the highest number of hyperglycemic episodes (β = 0.25, *p* = 0.002), the fewest hypoglycemic episodes (β = −0.18, *p* = 0.024), and the highest parental trait anxiety (β = 0.04, *p* < 0.001). Parents were found with moderate-to-high distress and anxiety, and their correlation also shows that there is an urgent need for suitable education of parents on managing the disease to improve quality of life and eliminate health risks to all involved.

## 1. Introduction

Diabetes mellitus (DM) is one of the most common diseases worldwide, with rapidly increasing incidence. The total number of people with DM is projected to double by 2030 to approximately one billion. Type 1 diabetes (DM1) is one of the most common chronic childhood diseases, with children forming 5% of the diabetic population [1]. DM seriously affects the lives of sufferers and their families. It is a long-term condition that could also be called a family disease due to the increased family support and stronger relationships required for its smooth management. The most difficult yet also the most important element of managing DM and discovering care and encouragement strategies is the acceptance of the disease both by the parents and by the sufferers themselves [2]. Equally important, depending on the age of the sufferer, is the promotion of trust by the parents in order to achieve self-efficacy [3]. Studies have shown that the higher the level of cooperation among carers (doctors, parents, and the sufferers themselves), the more positive the effects on DM-related factors such as glycemic control, quality of life, and emotional disorders [4].

Many studies have described the psychological state of parents of children with DM. One of the most recent is that by Holmström and colleagues [5], who detailed the stages through which parents pass following a DM diagnosis: (1) disbelief (denial of the disease), (2) lack of information and guilt, (3) learning to care, (4) normalization (return home to the new daily life), (5) uncertainty in care, and (6) reorganization (attempt to adapt to care).

In 2009, Βowes and colleagues presented the results of a qualitative study on a sample of parents of children with DM diagnosed 7–10 years previously [6]. The findings showed that a number of parents who had successfully adapted to diabetes management presented strong emotional responses during the interview when recalling the time of the diagnosis. A significant proportion of parents expressed strong feelings of grief at different points in the child’s development, high anxiety concerning the child’s future health, and a lack of coming to terms with the disease. High stress, sorrow, and anxiety about diabetes management also had a negative impact on couples’ relationships, even leading a small percentage to divorce [6].

The possible global impacts of coronavirus disease 2019 (COVID-19) on children and young adults during the SARS-CoV-2 pandemic have not yet been sufficiently studied. It has been reported that the disease is less prevalent in these age groups, forming approximately 1–2% of total cases. Moreover, a new-onset type 1 diabetes (T1D) associated with COVID-19 has been reported in children in the United Kingdom and the USA [7,8,9,10]. Although the pathophysiological changes in diabetic patients with COVID-19 are not yet clear, infection could lead to serious effects and even the appearance of comorbidities [11].

Type 1 diabetes is a long-term condition that requires complex clinical care and lifelong participation and can have a significant impact not only on the physical and mental but also on the emotional state of children, adolescents, and their carers. Increased levels of stress, anxiety, and depression have been reported during the pandemic, together with the fear of infecting or being infected by parents and relatives. Adolescents with T1D present increased levels of anxiety symptoms regarding both the disease itself and its management. Recent studies have also confirmed a high prevalence of symptoms of depression and anxiety in young people with T1D, noting that these can lead to poorer management of diabetes and glycemic control [12]. Moreover, patients with type 1 diabetes mellitus are considered a vulnerable population, but they do not appear to present increased frequency of COVID-19 infection. The basic hypothesis is that this is due to the peculiar immunological condition that leads to the destruction of beta cells and may control the role of the disease [13].

Taking the bibliographic references and data into account, it is necessary for healthcare professionals to pay attention to and prioritize educating less-educated parents in the management of their child’s disease. Support is also necessary in cases of low-socioeconomic status families, even including appropriate psychological or financial support or access to appropriate healthcare structures [14,15,16,17,18].

A key parameter that can change the management of the disease during the pandemic is a shift in family dynamics. From the beginning of the pandemic, this has been reflected in various aspects of the care of children with diabetes but not to a significantly higher degree. More specifically, the concern and fear of a potentially serious infection for the child constitutes a stressful situation, which, however, is not especially different from worrying about the appearance of any other infection. The suspension of school and extracurricular activities has resulted in children and adolescents spending more time at home, requiring greater attention from parents/carers. The restriction of activities and the closing of parks and recreation rooms has resulted in more sedentary habits and, consequently, lower daily energy expenditure. All these changes may significantly affect the glycemic control of the child, adding even more responsibilities and increasing the stress on parents/carers [19,20,21].

The aim of the present study was to investigate how parents of children, adolescents, and young adults with DM1 perceive their quality of life and the psychological burden they experience during a particularly stressful lockdown period such as that of the COVID-19 pandemic.

## 2. Materials and Methods

### 2.1. Study Design, Sample, and Participants 

A cross-sectional study was designed for the purposes of this research, and a purposive sampling design was used. The study sample consisted of parents of children, adolescents, and young adults with DM1 living in Greece. The study adhered to the Strengthening the Reporting of Observational studies in Epidemiology (STROBE) guidelines for cross-sectional studies (https://www.strobe-statement.org (accessed on 20 May 2022).

### 2.2. Research Tools

The study tools were the Parent Diabetes Distress Scale (PDDS) structured questionnaire for determining the quality of life of parents of children with DM1 and the Spielberger State/Trait Anxiety Inventory (STAI) for determining their psychological burden. The PDDS questionnaire (Behavioral Diabetes Institute, San Diego, CA, USA) comprises 20 questions evaluating how the parents of a child with diabetes feel. The questionnaire yields a total score (total of the scores of the 20 questions) measuring the distress of parents of children, adolescents, and young adults with DM and 4 subscale scores, each addressing a different kind of distress (personal distress, teen management distress, parent/teen relationship distress, healthcare team distress) [22]. The STAI consists of 40 sentences often used by people to describe themselves. The first 20 describe how they feel at the moment and the second 20 how they feel in their lives generally [23].

### 2.3. Data Collection

The study was conducted over 2 months (16 April–16 June 2021), and the data were collected via digital platform (Google Form) on the social network Facebook, on official Diabetes Associations websites, and on the official website of the Hellenic Diabetes Federation (HDF). The e-form contained information on the person responsible, the university and the aim of the present study, as well as the consent form. The data were extracted via Excel, ensuring their security and that of the participants’ consent forms.

### 2.4. Ethical Considerations

Ethical approval was obtained from the Research and Bioethics Committee (IRB; Hellenic Mediterranean University (Heraklion, Greece)—3510/8 March 2021). The participants in the study were informed about the study objectives, expected outcomes, and associated benefits and risks. They did not receive any compensation for their participation in the study. Written consent was received from the participants before they answered the questionnaire. 

### 2.5. Statistical Analysis 

The data were analyzed using SPSS software (IBM Corp. Released 2019, IBM SPSS Statistics for Windows, v.26.0, Armonk, NY, USA: IBM Corp.). Frequency distributions of the descriptive and clinical characteristics of parents and children with DM1 were estimated. The differences in the response distributions of the two scales used in the study, namely distress (PDSS) and anxiety (STAI), were established by chi-square test (homogeneity test). The shape of the score distribution was established using Blom’s Q-Q plot, while reliability coefficients were assessed by Cronbach’s α. Due to a slight asymmetry, parametric comparison tests were performed between the two scales and with the characteristics of parents and children. There followed a univariate Pearson correlation of the two scales and the characteristics of parents and children. Multiple linear regression was used to establish the β (unstandardized) coefficients of the anxiety and personal and clinical characteristics with parental quality of life (burden as distress). The significance level was 0.05.

## 3. Results

### 3.1. Basic Characteristics of Parents

Of the 110 parents of children, adolescents, and young adults with DM1 enrolled in the study, 79.1% were females, while the mean age of all was 44.4 years (±5.8) (Table 1). Regarding education, 52.8% held a university degree or postgraduate degree, 85.5% were married, 65.5% had two children, almost all (98.2%) had Greek nationality, and 75.5% declared a monthly income of up to EUR 1500. Regarding residence, 50.9% lived outside Crete, and the majority (84.5%) lived in an urban area. The characteristics of the children, adolescents, and young adults with DM1 (Table 2) showed that 50.9% were females, and the mean age of all was 13.0 years (±4.3) or from 3.5 to 26.0 years, including young adults. The mean disease duration was 5.7 years, ranging between 0.2 and 21.0. The majority (60.0%) had a treatment regimen of *multiple daily injections*, while half (50%) used a *glucose monitoring device* (*Libre*). Up to four hyperglycemic episodes a week were reported by 47.3%, and the same number of hypoglycemic episodes was reported by 60.9%. Generally, a higher frequency of hyperglycemic episodes is observed, but the difference is not significant (marginal homogeneity test, *p* = 0.091; results not shown in table/figure).

### 3.2. Parents’ Distress and Anxiety 

The Parent Diabetes Distress Scale (PDDS) for the previous month determines the score of its overall relation to quality of life and also four basic subscale scores (Table 3). The mean score was moderate or 2.42 (±0.76), scored from 1 (no burden or distress) to 5 (extreme), with a higher score denoting worse quality of life. Of the four subscales, the significantly highest score and therefore the highest Distress was found for *teen management distress*, while the lowest was found for *healthcare team* (*p* < 0.001). Their reliability was assessed with a Cronbach’s α of 0.773–0.919 (excellent). The Spielberger State/Trait Anxiety Inventory (STAI) of parents of children and adolescents with DM1 had a moderate to high score of 49.8 (±10.0) and 48.0 (±8.4), respectively, with a probable range of 20–80, with a higher score indicating higher anxiety. However, it should be noted that state anxiety (temporary anxiety) had a significantly higher mean score (*p* = 0.006). Their reliability was also assessed with a Cronbach’s α of 0.810 and 0.901 (excellent). It is also noted that 70.9% of respondents had high state anxiety, and 65.5% had high trait anxiety (situational anxiety). The highest frequency of moderate or high distress is also observed for *teen management distress* (77.3%) versus the lowest for *healthcare team* (14.5%). Moreover, 63.6% of parents had moderate-to-high general anxiety (results not shown in table/figure).

### 3.3. Characteristics, Quality of Life (Distress), and Anxiety

Finally, Table 4 presents a multivariate correlation using multiple linear regression of state/trait anxiety and characteristics of parents and children with DM1, with the quality of life determined by parental distress the previous month. Taking into account all the parameters tested by univariate correlations, the parents’ increased distress and consequently lower quality of life is not significantly correlated with their characteristics (*p* > 0.05), but it does appear to be significantly correlated with the highest number of hyperglycemic episodes (β = 0.25, *p* = 0.002), the fewest hypoglycemic episodes (β = −0.18, *p* = 0.024), and the highest parental trait anxiety (β = 0.04, *p* < 0.001). 

## 4. Discussion

The aim of the present study was to investigate the perceived quality of life of parents/carers of children and adolescents with DM1 through the distress they experience and their psychological burden or anxiety due to their child’s disease during the COVID-19 pandemic. To summarize, the following findings emerged: (a) parents had moderate levels of Distress, where higher distress indicates worse quality of life, with *teen management distress* being the significantly highest and *healthcare team* the lowest (*p* < 0.001); (b) approximately two-thirds of parents had moderate or high overall distress; (c) moderate-to-high levels of state and trait anxiety were found, with significantly higher levels of state anxiety (*p* = 0.006), while over two-thirds of parents had high levels; (d) parental state anxiety is correlated with lower child age, lower monthly income, fewer years duration of their child’s disease, and treatment with multiple daily injections, while trait anxiety is correlated with a lower level of education (*p* < 0.05); and (e) increased parental distress and consequently poorer quality of life is not significantly correlated with parents’ characteristics, but it is significantly correlated with the highest number of hyperglycemic episodes, the fewest hypoglycemic episodes, and the highest parental trait anxiety (*p* < 0.05). 

Type 1 diabetes is one of the most common diseases in children and adolescents, with a prevalence of 1 in 400 children and adolescents, involving a multitude of changes to their lives and those of their families [24]. They are forced to follow treatment regimens that include insulin injections, physical exercise, and healthy dieting adapted to avoid hyperglycemic or hypoglycemic episodes, averting the risk to their life and health. 

In this context, their families and especially their parents regularly experience, on a daily basis, stress and anxiety about their children’s health. In their recent review of the literature on parental stress and symptoms of stress and depression associated with self-efficacy in children with DM1, Bassi and colleagues [24] found several interesting results. They showed that parents experience relatively high levels of stress, depression, and symptoms associated with the management of their children’s disease and also with their own parental self-efficacy. They also demonstrated that parental stress predicts or is correlated with worsening control of children’s hemoglobin A1c (HbA1c) levels, while parental diabetes-specific distress is a predictor of increased symptoms of depression in the children and adolescents. Similarly, in the present study, increased parental distress (as worsening quality of life) is significantly correlated with the highest number of hyperglycemic episodes, the fewest hypoglycemic episodes, and also the basic prognostic factor of parental trait anxiety (*p* < 0.05). In order to manage both the disease itself and the parents’ anxiety, which affects their quality of life, some studies propose high parental self-efficacy, associated with better monitoring, and direct interventions in the control and regulation of HbA1c in the children and adolescents [16]. In the study by Alessi and colleagues, it was found that periods of increased social stress, such as the period of lockdown due to the pandemic, may increase symptoms of anxiety, burden, and mental health disorders in some caregivers of children and adolescents with diabetes. Understanding the burden these families may experience during the stressful situation leads to the development of new strategies to support these caregivers [25]. These interventions should be aimed at fostering social support, improving diabetes management, and decreasing perceived stress, alleviating parents’ anxiety and focusing on increasing their self-efficacy. Streisand and colleagues [26] and Luo and colleagues [14] proposed that healthcare providers and educators should help to manage parental anxiety and depression, increasing their self-efficacy, and that where depression may exist, the person should be referred to a mental health specialist. 

In their systematic review, Whittemore and colleagues [27] attempted to investigate the prevalence of parental psychological distress and its relationship with parents’ health. They found a prevalence of parental distress ranging from 10% to 74% in the studies reviewed, with an average of 33.5% of parents reporting distress at diagnosis of the disease and 19.0% reporting distress 1 to 4 years after diagnosis. Moreover, parental distress was correlated with high report of stress and depression by the children themselves, problematic child behavior, and lower quality of life of the child. The authors concluded from their review of the literature that parental distress had a negative impact on the management of their children’s disease. It is noted that in the present study the prevalence of moderate or high parental distress was 63.6%, which is within the range determined by Whittemore and colleagues [27], although it was not correlated to the characteristics of the children or to their disease. On the contrary, parental anxiety was correlated with lower child age, fewer years’ duration of their child’s disease, or a treatment regimen of multiple daily injections. Besides the burden on the parents of children and adolescents with DM1, research should also focus on the burden on the children themselves as patients and its consequences on the management of the disease and their daily lives [15,28,29]. In other words, lower child anxiety may result in lower parental anxiety. However, parent diabetes stress was associated with more frequent blood glucose monitoring and better self-care behavior [30].

In the present study, the frequency of moderate or high parental distress was around 64%, while increased anxiety (state or trait) was >65%. Bousdoglou [31] studied state/trait anxiety (STAI) in 129 parents and found moderate mean levels (53.2 and 47.9, respectively), roughly similar to the findings of the present study, while there was a significant negative correlation with the World Health Organization Quality of Life (WHOQOL) subscales. Similarly, Hilliard and colleagues [32] recorded, from the reports of the parents, that an increase in their general and pediatric anxiety is correlated with the appearance of problematic behavior by the children. Arafa and Alwakeel [33] noted increased stress in 36% of parents, which is significantly higher than the control group in their comparisons, and underlined that those parents are at increased risk of anxiety that affects their quality of life. The present study showed that of the main causes, with every reservation due to the cross-sectional methodology used, increased parental distress is significantly affected by the highest number of hyperglycemic and fewest hypoglycemic episodes. Among the basic causes was parents’ fear of possible hypoglycemic episodes, which negatively affected both children and parents. Paradoxically, hypoglycemic episodes have been found to be associated with better quality of life or at least do not appear to increase parental anxiety, as do hyperglycemic episodes (obviously busy them less). Furthermore, other studies have shown evidence of parents’ efforts to avoid hypoglycemia drives to opposite results, leading to poorer glycemic control. This must draw attention to the urgent need to educate parents in order to reduce their anxiety [14,34].

Overall, as a general observation, no particular differences were noted in the relationship between the distress experienced by the parents of children with DM1 and their psychological burden or anxiety about their child’s disease between the non-pandemic period and the period of the COVID-19 pandemic. 

Although no strong scientific data have yet been published, these groups of asymptomatic patients with uncontrolled diabetes may be at risk of developing COVID-19 more easily. This could be triggered by the increase in stress hormones and arterial tension, which may also arise due to the pandemic conditions. Psychological support of these patients could therefore play a key role in their protection. Diabetes patients should be reassured that their doctors are accessible and available at all times via phone or email [35,36]. Garg and colleagues [37] discovered that during the COVID-19 pandemic, the use of telemedicine technologies for the management of new-onset T1D in children and adults is effective and feasible.

## 5. Limitations

The methodological design of the present study aimed to demonstrate the levels of anxiety and distress in parents of children with DM1. Nevertheless, the basic limitation of the current study was the selection of the sample and the implementation of the research during the periodic and repeatable lockdowns. However, the final assessment lacks comparison with other diseases of children/adolescents, which may also cause increased parental anxiety and distress, affecting their overall family quality of life. Moreover, the current conditions of the Greek healthcare system, the prohibitions or reservations, and the care necessary at the individual or collective level due to the pandemic and its restriction measures are an obstacle to any study. Research, therefore, including the present study, often has to be conducted remotely; to overcome the mentioned obstacles, the authors used the electronic data collection and mainly used the official websites of their associations as well as social media, which may give a certain bias to the collection of sensitive information (e.g., the absence of an interview). 

## 6. Conclusions

The present study aimed to investigate how parents of children, adolescents, and young adults with DM1 perceive their quality of life and the psychological burden they experience during a particularly stressful lockdown period, such as that of the COVID-19 pandemic. The parents were found to have moderate levels of distress and moderate-to-high levels of state and trait anxiety, while parental state anxiety was not correlated with child age, monthly income, duration of their child’s disease, or a treatment regimen of multiple daily injections. However, increased parental distress was significantly correlated with the highest number of hyperglycemic episodes, the fewest hypoglycemic episodes, and the highest parental trait anxiety. It is recommended that healthcare professionals provide appropriate education to less-educated parents in the management of their child’s disease and also that parents are provided the appropriate psychological or financial support or access to appropriate healthcare structures.

## Figures and Tables

**Table 1:** General characteristics of 110 participating parents of children with DM1.

			*n*	%
Gender	*Male*		23	20.9
	*Female*		87	79.1
Age, *years*	*Mean age (stand. dev.) [min., max.]*		44.4 (5.8) [31, 58]	
Education	*Graduated from*	*Primary School*	4	3.6
		*Secondary School*	48	43.6
		*Higher Education*	38	34.5
		*MSc, Ph.D.*	20	18.3
Marital status	*Married, With a partner*		94	85.5
	*Unmarried, divorced*		16	14.5
Number of children	*One*		20	18.2
	*Two*		72	65.5
	*Three*		14	12.7
	*Four*		4	3.6
Nationality	*Greek*		108	98.2
	*Other*		2	1.8
Monthly income, EUR	*<500*		15	13.6
	*500–1000*		25	22.7
	*1001–1500*		43	39.2
	*1501–2000*		11	10.0
	*>2000*		16	14.5
Place of residence	*Crete*		56	50.9
	*Central Greece and Aegean Islands*		32	29.1
	*Northern Greece*		22	20.0
Area of residence	*Rural*		17	15.5
	*Urban*		93	84.5

**Table 2:** Characteristics of 110 children/adolescents with DM1 of participating parents.

		*n*	%
Gender	*Male*	54	49.1
	*Female*	56	50.9
Age, *years*	*Mean age (stand. dev.) [min., max.]*	13.0 (4.3) [3.5, 26.0]	
Disease duration, *years*	*Mean years (stand. dev.) [min., max.]*	5.7 (4.5) [0.2, 21.0]	
Treatment regimen	*Insulin pump*	44	40.0
	*Multiple daily injections*	66	60.0
Blood sugar level monitoring	*Glucometer strips*	19	17.3
	*Glucose monitoring device (Libre)*	55	50.0
	*Continuous Glucose Monitor*	36	32.7

**Table 3:** Subscale scores of Parent Diabetes Distress Scale (PDDS) the previous month and Spielberger State/Trait Anxiety Inventory (STAI) of parents of children and adolescents with DM1.

	Mean	Stand. Dev.	Median	Min	Max	Cronbach’s α
Parent Diabetes Distress Scale ^a^						
Distress of parents of children and adolescents with DM1	2.42	0.76	2.38	1.00	4.75	0.919
Personal distress	2.34	0.89	2.17	1.00	4.83	0.814
Teen management distress	3.02	0.99	3.00	1.00	5.00	0.773
Parent/teen relationship distress	2.41	0.82	2.38	1.00	4.63	0.829
Healthcare team distress	1.49	0.93	1.00	1.00	5.00	0.883
State/Trait Anxiety Inventory ^b^						
State anxiety	49.8	10.0	51.0	29	70	0.846
Increased levels (45+)	n = 78 or 70.9%					
Trait anxiety	48.0	8.4	48.0	30	69	0.810
Increased levels (45+)	n = 72 or 65.5%					
Overall anxiety	97.8	17.2	100.5	60	136	0.901

^a^ Scoring from 1 = none to 5 = extreme. Higher score indicates higher distress. Friedman test across subscales, *p* < 0.001. ^b^ Higher score indicates higher anxiety. Student’s test between State and Trait Anxiety, *p* = 0.006.

**Table 4:** Multiple linear regression of state and trait anxiety and characteristics of parents and children with DM1, with quality of life determined by parental distress the previous month.

	Quality of Life (Higher Score of Distress Indicates Worse QoL)			
*Prognostic Factors*	β Coefficients	95%CIs		p-value
***Parents***				
**Gender** (1: male, 2: female)	−0.18	−0.48	0.13	0.250
**Age** (years)	−0.02	−0.04	0.01	0.214
**Education** (1: primary, 2: secondary, 3: higher, 4: M.Sc., Ph.D.)	−0.01	−0.17	0.16	0.963
**Marital status** (1: Married, With partner, 2: Unmarried, Divorced)	−0.06	−0.39	0.28	0.741
**Number of children**	−0.05	−0.23	0.13	0.554
**Monthly income, EUR** (1:<500, 2:500–1000, 3: 1001–1500, 4: 1501–2000, 5:>2000)	−0.01	−0.13	0.10	0.813
**Area of residence** (1: rural, 2: urban)	−0.24	−0.59	0.11	0.170
***Children with DM1***				
**Age** (years)	0.04	−0.01	0.08	0.090
**Duration of disease** (years)	0.02	−0.02	0.05	0.395
**Treatment regimen** (1: insulin pump, 2: multiple daily injections)	0.04	−0.22	0.30	0.765
**Hyperglycemic episodes** (1:0–1, 2: up to 4, 3: up to 10, 4: >10)	0.25	0.09	0.40	0.002
**Hypoglycemic episodes** (1:0–1, 2: up to 4, 3: up to 10, 4: >10)	−0.18	−0.34	−0.02	0.024
**Parental State Anxiety** (higher score indicates higher anxiety)	0.01	−0.01	0.03	0.227
**Parental Trait Anxiety** (higher score indicates higher anxiety)	0.04	0.02	0.07	<0.001
*R^2^ (adjusted)*	0.499 (0.425)			

## Data Availability

The data presented in this study are available on request from the corresponding author. The data are not publicly available due to the ethical commitment.
